# The Self-Pleasantness Judgment Modulates the Encoding Performance and the Default Mode Network Activity

**DOI:** 10.3389/fnhum.2016.00121

**Published:** 2016-03-18

**Authors:** Marcela Perrone-Bertolotti, Melanie Cerles, Kylee T. Ramdeen, Naila Boudiaf, Cedric Pichat, Pascal Hot, Monica Baciu

**Affiliations:** ^1^Laboratoire de Psychologie et Neurocognition (LPNC), University Grenoble AlpesGrenoble, France; ^2^Laboratoire de Psychologie et Neurocognition (LPNC), Centre National de la Recherche Scientifique (CNRS), UMR 5105Grenoble, France; ^3^Laboratoire de Psychologie et Neurocognition (LPNC), University Savoie Mont BlancChambéry, France; ^4^School of Psychology, University of OttawaOttawa, ON, Canada

**Keywords:** default-mode network, memory encoding performance, pleasantness judgment, self-related emotion, precuneus, posterior cingulate cortex

## Abstract

In this functional magnetic resonance imaging (fMRI) study, we evaluated the effect of self-relevance on cerebral activity and behavioral performance during an incidental encoding task. Recent findings suggest that pleasantness judgments reliably induce self-oriented (internal) thoughts and increase default mode network (DMN) activity. We hypothesized that this increase in DMN activity would relate to increased memory recognition for pleasantly-judged stimuli (which depend on internally-oriented attention) but decreased recognition for unpleasantly-judged items (which depend on externally-oriented attention). To test this hypothesis, brain activity was recorded from 21 healthy participants while they performed a pleasantness judgment requiring them to rate visual stimuli as pleasant or unpleasant. One hour later, participants performed a surprise memory recognition test outside of the scanner. Thus, we were able to evaluate the effects of pleasant and unpleasant judgments on cerebral activity and incidental encoding. The behavioral results showed that memory recognition was better for items rated as pleasant than items rated as unpleasant. The whole brain analysis indicated that successful encoding (SE) activates the inferior frontal and lateral temporal cortices, whereas unsuccessful encoding (UE) recruits two key medial posterior DMN regions, the posterior cingulate cortex (PCC) and precuneus (PCU). A region of interest (ROI) analysis including classic DMN areas, revealed significantly greater involvement of the medial prefrontal cortex (mPFC) in pleasant compared to unpleasant judgments, suggesting this region’s involvement in self-referential (i.e., internal) processing. This area may be responsible for the greater recognition performance seen for pleasant stimuli. Furthermore, a significant interaction between the encoding performance (successful vs. unsuccessful) and pleasantness was observed for the PCC, PCU and inferior frontal gyrus (IFG). Overall, our results suggest the involvement of medial frontal and parietal DMN regions during the evaluation of self-referential pleasantness. We discuss these results in terms of the introspective referential of pleasantness judgments and the differential brain modulation based on internally- vs. externally-oriented attention during encoding.

## Introduction

Internally-oriented tasks are generally associated with a disengagement of attention from the external environment to internal thoughts, resulting in a superficial involvement in processing external stimuli with poor task performance (Smallwood and Schooler, 2006). Internally-oriented tasks depend, at least partially, on regions belonging to the default mode network (DMN; Harrison et al., 2008; Andrews-Hanna et al., 2014b). The DMN (see Gusnard and Raichle, 2001; Raichle et al., 2001; Raichle and Snyder, 2007; Raichle, 2010; Snyder and Raichle, 2012) is composed of midline anterior medial prefrontal cortex (mPFC) and posterior (retrosplenial; posterior cingulate cortex/precuneus, PCC/PCU) cortices, as well as lateral regions including the inferior parietal lobule (IPL), superior frontal cortex (SPC) and lateral temporal cortex (LTC). Although the exact functional role of the DMN is not fully understood (Fox et al., 2005; Andrews-Hanna et al., 2014a), it appears that the DMN plays a role in attentional demands and executive functions (Smallwood et al., 2012; Andrews-Hanna et al., 2014a). Indeed, previous studies suggest that the level of DMN activity could reflect various degrees of a subject’s involvement in processing external stimuli (Buckner et al., 2008). Specifically, increased DMN activity would reflect poor attention toward external items and low behavioral performance; whereas decreased DMN activity would rather reflect a significant amount of externally-oriented attention, leading to higher performances (Ossandón et al., 2011; Spreng, 2012). However, a few studies have demonstrated an opposite and paradoxical effect, when DMN was significantly activated and associated with a high level of performance (Kelley et al., 2002; Leshikar and Duarte, 2012; Maillet and Rajah, 2014). This situation can be typically observed during tasks requiring a significant amount of self-reference that depend on introspective mental thoughts. When a task requires internally-oriented attention, the DMN involvement may be considered task-successful (e.g., Spreng et al., 2010), as an increase in activity reflects an increase in internally-oriented processes helpful in performing the task. This situation underlines the importance of distinguishing between task-relevant internally-oriented thoughts that lead the DMN to be task-successful (e.g., Maillet and Rajah, 2014) from task-irrelevant spontaneous internal thoughts that lead the DMN to be task-unsuccessful (e.g., Fox et al., 2005; Kim, 2011). In other words, tasks requiring self-relevance and internally-oriented attention recruit the DMN. Within this framework, Maillet and Rajah (2014) showed that performing an encoding task based on the judgment of self-pleasantness (i.e., whether a stimulus feels subjectively pleasant or unpleasant), requires self-generated internal processes rather than processing external cues. When participants make a pleasantness judgment, they must depend on autobiographical processes, and semantic and episodic memories related to that emotional judgment. In fact, studies on emotional memory have highlighted that processes recruited by successfully-encoded items vary according to the valence (pleasant or unpleasant) of the given judgment (Kensinger and Schacter, 2008; Mickley and Kensinger, 2008; Ritchey et al., 2011). Specifically, pleasant or positive judgments tend to benefit from semantic, episodic and self-referential internally-oriented processing, whereas unpleasant or negative judgments tend to benefit from perceptual, low-level and externally-oriented processing (Mickley and Kensinger, 2008). Given that the pleasantness task induces self-relevant thoughts and internally-oriented attention, this kind of task should enhance the processing and encoding of pleasant judgments while impairing the encoding of unpleasant stimuli.

In this study, we evaluated to what extent the pleasantness judgment modulates cerebral activity and memory recognition using an incidental memory encoding task. We tested these interactions using both a region of interest (ROI) analysis, focused on three key DMN regions, as well as a whole brain analysis. We expected to find support for our hypothesis that pleasant judgments require a significant involvement of the DMN and internally-oriented attention thus leading to better encoding performance, whereas unpleasant judgments require a lesser involvement of the DMN and have poorer encoding performance.

## Materials and Methods

### Participants

Twenty-one healthy adults (10 females, mean age = 27.33 years, *SD* = 3.89 years, age range 19–34 years) participated in the study. All participants were right-handed according to the Edinburgh Handedness Inventory (Oldfield, 1971), native French speakers and had no history of neurological and psychiatric disorders. They gave their informed written consent to participate to the experiment. The study was approved by the local Ethics Committee (CPP no. 09-CHUG-14, April 6th, 2009).

### Tasks, Stimuli and Paradigm

**Figure 1:**
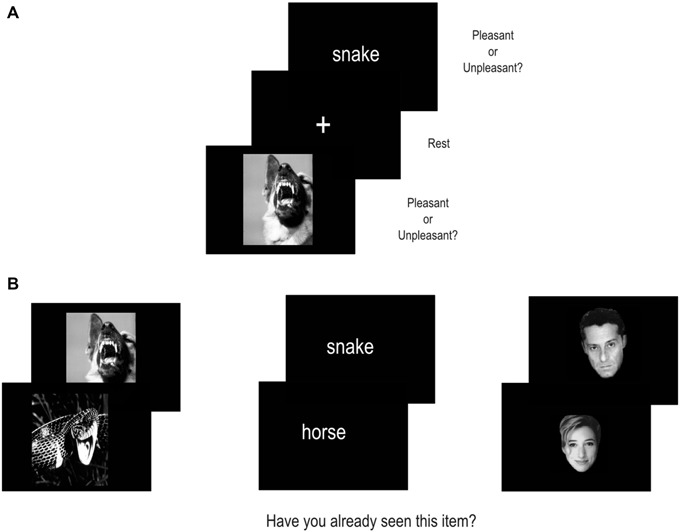
**Illustration of the experimental procedure. (A)** shows the encoding task performed inside the MR scanner. **(B)** shows the recognition task performed outside the MR scanner.

Each participant performed two tasks, an incidental encoding memory task inside the MR scanner followed by a recognition task outside the MR scanner (see Figure 1 for an illustration of the procedure).

#### Encoding Task during fMRI

##### Procedure

During the incidental encoding task, participants were instructed to perform a pleasantness judgment, that is, to decide whether a stimulus was pleasant or unpleasant and to provide manual responses using the index (for pleasant) and the middle finger (for unpleasant) of the right hand. To avoid the use of memory strategies, participants were not explicitly instructed to memorize items and were not informed of the subsequent memory recognition task. Furthermore, participants were informed that there were no correct or incorrect responses and that their judgment should be based on their own instantaneous appreciation of the pleasantness. We chose to not include a neutral condition, in order to force participants to search for subjective self-related emotional states (either pleasant or unpleasant) associated with stimulus attributes. All participants underwent a training session with different stimuli, before entering into the MR scanner.

##### Stimuli

We used 180 visual mixed stimuli representing verbal (words) and non-verbal (unfamiliar faces; pictures) material. Stimuli were chosen according to their objective emotional valence, half of them being considered pleasant and the other half unpleasant. Stimuli objectively rated as pleasant and unpleasant, were used to maximize the chance of obtaining an equivalent number of pleasant and unpleasant judgments during the encoding task. Words were French concrete familiar nouns composed of 6–7 letters (average frequency = 43.84 according to New et al., 2001) selected from Bonin et al. (2001) normative battery. Their emotional valence was determined according to Bonin et al. (2001) classification. They were written in white “Courier New” font, size 14 and centered on a black background. Unfamiliar faces were selected according to their objective emotional valence from an *in-house* database; they were displayed in a gray scale on a black background. Pictures were selected from the International Affective Picture System (IAPS; Lang et al., 1999), based on their objective emotional valence (scores for negative pictures between 1 and 4, and scores for positive pictures between 6 and 9) and displayed identically to faces.

Each stimulus was presented for 3.5 s and was followed by a 0.5 s of fixation cross, the total trial duration being 4 s (Figure 1). Stimuli were presented via E-prime Software (Psychology Software Tools Inc., Pittsburgh, PA, USA) running on a PC computer and displayed at the center of a black screen. They were transmitted into the MR imager by means of a video projector (Epson EMP 8200), a projection screen and a mirror centered above the participant’s eyes.

##### Functional MRI paradigm

We used a mixed block-event related paradigm with previous optimization of stimulus onset (Friston et al., 1999). The 180 stimuli were presented along six runs, each run including one block of 30 items (i.e., 10 pictures, 10 faces and 10 words) in which all stimuli of the same type were presented together, followed by a rest condition of 20 s (total duration per run: 140 s). To stabilize the MR magnetic field, the first five “dummy” scans were discarded from the subsequent analyses. Overall, 288 functional volumes were acquired for a total duration of 18 min.

##### MR acquisition

Experiments were performed in a whole-body 3T MR scanner (BrukerMedSpec S300). Functional images were obtained using a T2*-weighted, gradient-echo, echoplanar imaging (EPI) sequence with whole-brain coverage (Repetition time = 3 s, spin echo time = 40 ms, flip angle = 77). Thirty-nine axial slices parallel to the antero-posterior commissural plane were acquired in interleaved order (3 × 3 mm in plane resolution with a slice thickness of 3.5 mm). A B0 fieldmap was also acquired from two gradient echo data sets with a standard 3D FLASH sequence (ΔTE = 9.1 ms). In addition, a high-resolution T1-weighted whole-brain structural image was acquired for each participant (MP-RAGE, volume of 256 × 224 × 176 mm^3^ with a resolution of 1.33 × 1.75 × 1.37 mm^3^).

#### Recognition Task Outside the MR Imager

The recognition task was performed 1 h later the functional magnetic resonance imaging (fMRI) examination, outside the MR imager, on a computer. Participants were shown the 180 test stimuli mixed with 180 new stimuli, and were instructed to indicate the stimuli previously seen during the encoding task. The new stimuli were selected from the same databases as the test stimuli. Given that we were interested to evaluate the effect of the subjective pleasantness judgment on the encoding performance and on DMN activation, we performed a *post hoc* classification of trials in Pleasant and Unpleasant, based on each subject’s responses during the incidental encoding task. The encoding performance was based on recognition accuracy measured during the subsequent recognition task. The successful encoding (SE) condition corresponded to subsequently correctly recognized events whereas the unsuccessful encoding (UE) condition corresponded to subsequently forgotten events. Finally, four conditions of interest have been included, Pleasant-SE, Pleasant-UE, Unpleasant-SE and Unpleasant-UE.

### Data Processing

#### Behavioral Analyses

In order to assess the effect of the *Pleasantness judgment* (Pleasant, Unpleasant) on the *Encoding performance* (SE, UE), 2 × 2 within-subject analyses of variance (ANOVA) were conducted on the response rates (%CR Correct responses) and the reaction times (RTs, in milliseconds) measured during the encoding task. As described previously, the *encoding performance* was based on response accuracy in the recognition task, whereas *pleasantness* was based on participant’s subjective ratings during the incidental encoding task.

#### fMRI Data Processing

##### Spatial pre-processing

For each participant, functional images were first, time-corrected (slice timing using the middle slice as a reference). All volumes were realigned to correct for the head motion using rigid body transformations. Unwrapping was performed using the individually acquired fieldmaps to correct for the interaction between head movements and EPI distortions (Andersson et al., 2001). The T1-weighted anatomical volume was co-registered to mean images created by the realignment procedure and was normalized to the MNI space using a trilinear interpolation. The anatomical normalization parameters were then used for the normalization of functional volumes. All functional images were subsequently smoothed using a 6 mm full-width at half maximum Gaussian kernel to improve the signal-to-noise ratio and to compensate for the anatomical variation between individual brains.

##### Statistical analyses

For each participant, four conditions were modeled by means of the General linear model (Friston et al., 1994): Pleasant-SE, Pleasant-UE, Unpleasant-SE and Unpleasant-UE. Six realignment parameters were also included in the design matrix as covariates of no interest. The blood-oxygen-level dependence (BOLD) response for each event was modeled using a canonical hemodynamic response function (HRF). Before estimation, a high pass filtering with a cutoff period of 128 s was applied. Beta weights associated with the modeled HRF responses were then computed to fit the observed BOLD signal time course in each voxel for each of the four conditions.

Two types of statistical analyses were performed: a ROI analysis and a whole brain analysis. Both analyses had the following three goals: (a) identification of cerebral regions underlying the subjective pleasantness judgment (Pleasant, Unpleasant); (b) identification of cerebral regions underlying the encoding performance (i.e., encoding performance SE, UE); and (c) identification of a possible interaction effect between the pleasantness judgement and encoding performances.

ROI analyses were performed by using *a priori* ROI masks for three DMN regions, as proposed by Fox et al. (2005). These regions were, bilaterally, the mPFC, the PCC/PCU and the Lateral Parietal Cortex (LPC). Specifically, we retained all activated voxels included within a 6 mm radius around the MNI peak of activation reported by Fox et al. (2005) in the left and in the right hemispheres (RHs) (i.e., mPFC: ±1 48 −1; PCC/PCU: ±5 −52 40 and LPC: ±45 −70 35). MarsBar Software^1^ was used to build ROIs. For each ROI and each participant, the percentage of MR signal change was measured and the corresponding values were included into a 3 × 2 × 2 within-subject ANOVA. This analysis allowed us to evaluate the effect of the pleasantness judgment (Pleasant, Unpleasant) and encoding performance (SE, UE) on the activity of the three DMN regions. A separate ANOVA was performed for the left and for the RH.

A subsequent whole brain analysis was performed. To draw population-based inferences (Friston et al., 1998), a second-level random effect group analysis was carried-out. An ANOVA was performed based on individual analyses by means of a flexible-factorial design following the guidelines of Glascher and Gitelman (2008). This ANOVA modeled the subjective pleasantness judgment (Pleasant, Unpleasant) and encoding performance (SE, UE) as within-subject factors in order to test the interaction between them. The significance value was set at *p* < 0.001, uncorrected for each contrast (height threshold *T* = 3.55) with a voxel cluster extent estimated for each contrast with a Monte Carlo simulation (using the REST toolkit, Song et al., 2011). These voxel cluster extended (*k*) correspond to: *k* > 60 for the main effect of pleasantness judgmement; *k* > 50 for the main effect of encoding performance and *k* > 55 for the interaction between pleasantness judgmement and encoding performance. Similar to the previously mentioned ROI analysis, this ANOVA allowed us to evaluate the effects of the pleasantness judgement, encoding performance and the relationship between both factors on cerebral activity in all brain regions (outside and within the DMN).

## Results

### Behavioral Results

The analysis conducted on RTs showed no significant difference (*F*_(1,20)_ = 2.76, *p* = 0.11, $\eta_{p}^{2}$ = 0.12) between Pleasant (*M* = 53%, *SD* = 7%) and Unpleasant (*M* = 47%, *SD* = 7%) ratings. The analysis conducted on performances showed a significant main effect of encoding (*F*_(1,20)_ = 8.81, *p* < 0.05, $\eta_{p}^{2}$ = 0.30), indicating that the majority of stimuli were Successfully encoded vs. unsuccessfully encoded (*M* = 59%, *SD* = 14%). The interaction between pleasantness ratings and encoding performance was significant (*F*_(1,20)_ = 7.04, *p* < 0.05, $\eta_{p}^{2}$ = 0.26). The decomposition of the interaction showed (*F*_(1,20)_ = 9.39, *p* < 0.05) that SE items were more often rated as Pleasant (*M* = 33%, *SD* = 10%) than Unpleasant (*M* = 27%, *SD* = 8%); this difference was absent for UE items (*F* < 1). The analysis conducted on RTs revealed a significant main effect of pleasantness. Participants were faster (*F*_(1,20)_ = 8.87, *p* < 0.05, $\eta_{p}^{2}$ = 0.30) to judge items as Pleasant (*M* = 1383 ms; *SD* = 297 ms) than Unpleasant (*M* = 1485 ms; *SD* = 343 ms). No significant effect was found, neither for encoding performance (*F*_(1,20)_ = 1.64, *p* = 0.21, $\eta_{p}^{2}$ = 0.08) nor for the interaction between pleasantness ratings and encoding performance (*F* < 1).

### Functional MRI Results

#### ROI Analysis with DMN Regions

**Figure 2:**
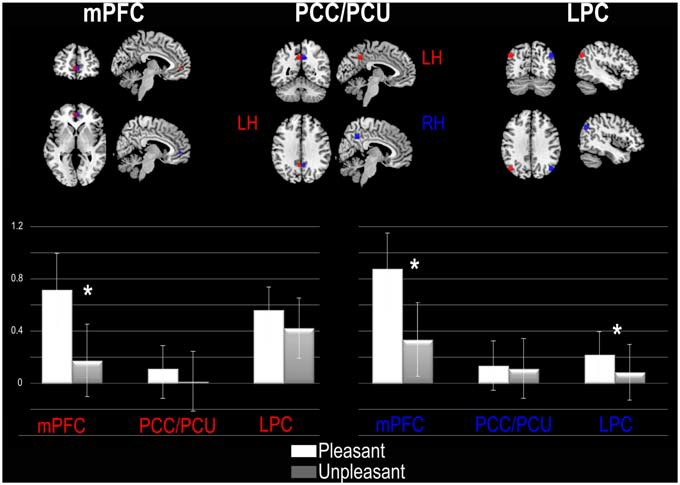
**ROI analysis based on default mode network (DMN) regions reported by Fox et al. (2005).** For each DMN region the percent of MR (%MR) signal change is presented for the left (red) and right (blue) hemisphere. Abbreviations: mPFC, medial Prefrontal Cortex; PCC/PCU, Posterior Cingulate Cortex/Precuneus; LPC, Lateral Parietal Cortex; LH, Left Hemisphere; RH, Right Hemisphere. Significant statistical differences are represented by asterisk.

As illustrated in Figure 2, the ANOVA performed at the left hemisphere (LH) shows a significant main effect of Pleasantness judgment (*F*_(1,20)_ = 7.86, *p* = 0.01) with a higher % of MR signal change for Pleasant (*M* = 0.80% MR, *SD* = 1.14% MR) than for Unpleasant (*M* = 0.54% MR, *SD* = 1.02% MR) trials. The ANOVA also showed a significant interaction between the DMN regions and Pleasantness (*F*_(2,40)_ = 9.28, *p* = 0.0004). Planned comparisons revealed that the significant difference between Pleasant and Unpleasant conditions was only observed for the mPFC, with greater signal change during the Pleasant (*M* = 0.71% MR, *SD* = 2.2% MR) than during the Unpleasant condition (*M* = 0.17% MR, *SD* = 1.2% MR). The RH ANOVA revealed a significant main effect of the DMN region (*F*_(2,40)_ = 3.38, *p* = 0.04) with greater involvement of the mPFC (*M* = 0.61% MR, *SD* = 0.92% MR) than the PCC/PCU (*M* = 0.12% MR, *SD* = 0.96% MR) and the PLC (*M* = 0.15% MR, *SD* = 1.31% MR). We also obtained a significant main effect of Pleasantness (*F*_(1,20)_ = 16.78, *p* = 0.0005) with greater signal change during the Pleasant (*M* = 0.41% MR, *SD* = 1.27% MR) than Unpleasant condition (*M* = 0.18% MR, *SD* = 1.05% MR). A significant interaction between the DMN regions and Pleasantness was once again observed (*F*_(2,40)_ = 9.43, *p* = 0.0004), revealing a significant difference between the Pleasant and Unpleasant conditions for the mPFC and PLC. For the mPFC, the Pleasant judgments (*M* = 0.87% MR, *SD* = 1.29% MR) induced greater signal change than Unpleasant judgments (*M* = 0.34% MR, *SD* = 1.27% MR). Similarly, for the right PLC, the Pleasant judgment (*M* = 0.21, *SD* = 0.82) induced greater signal change than Unpleasant judgments (*M* = 0.08% MR, *SD* = 0.98% MR). No main effect or interaction was observed for the encoding performance.

#### Whole Brain Analysis

**Figure 3:**
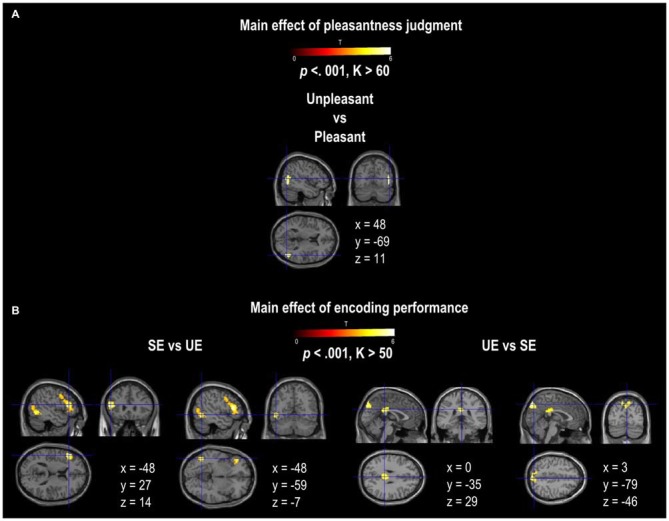
**Activation provided by the random-effect group analyses. (A)** shows the activation for the main effect of pleasantness judgment (Pleasant vs. Unpleasant; Unpleasant vs. Pleasant). **(B)** shows the activation for the main effect of encoding performance (SE vs. UE and UE vs. SE). The activation is projected onto 2D anatomical slices in axial, coronal, and sagittal orientations, MNI coordinates are presented. The color scale indicates the *T* value of the activation (height threshold *T* = 3.55, *p* < 0.001 uncorrected) with a voxel cluster extent estimated for each contrast with a Monte Carlo simulation. Abbreviations: SE, Successful Encoding; UE, Unsuccessful Encoding.

**Figure 4:**
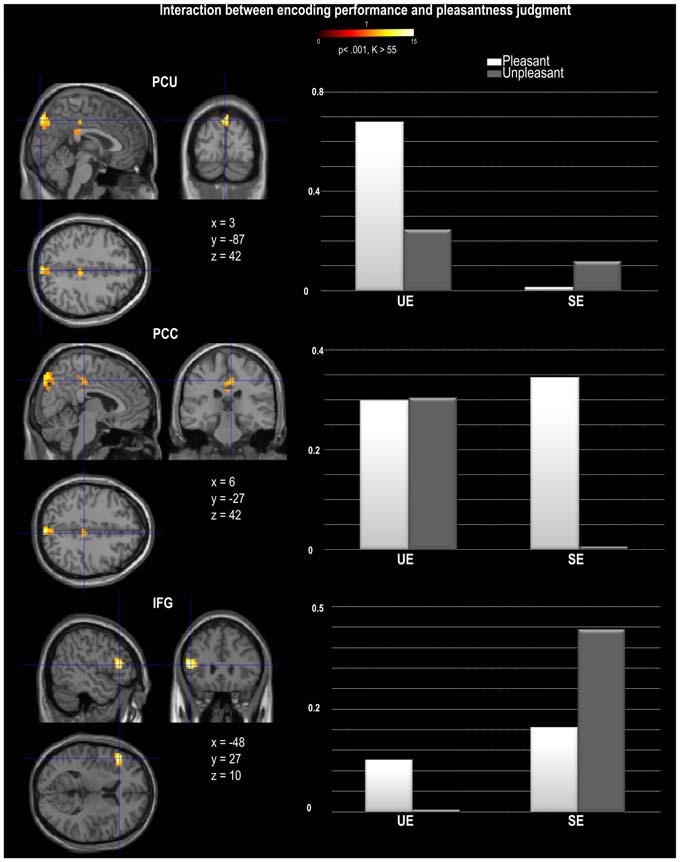
**Activation provided by the random effect group analysis on the statistical interaction between pleasantness judgment and encoding performance.** The direction of the interaction was represented by the estimates parameters for each experimental condition and each region, PCU, PCC and IFG (inferior frontal gyrus). The activation is projected onto 2D anatomical slices in axial, coronal, and sagittal orientations, MNI coordinates are presented. The color scale indicates the *T* value of the activation (height threshold *T* = 3.55, *p* < 0.001 uncorrected) with a voxel cluster extent estimated with a Monte Carlo simulation (*K* = 55). Abbreviations: SE, Successful Encoding; UE, Unsuccessful Encoding.

A main effect of Pleasantness was observed for the right inferior and middle temporal gyri, which were both more involved in Unpleasant than in Pleasant judgments (as illustrated in Table 1, Figure 3A). No region was found to be more significantly activated during Pleasant than Unpleasant trials. A main effect of the encoding performance was also found for the left inferior frontal gyrus (IFG, including pars triangularis and orbitalis), left precentral gyrus, and left inferior and middle temporal gyri which were more activated in SE than in UE (see Table 1, Figure 3B). Contrarily, the bilateral PCU and bilateral PCC, two DMN structures, were more activated during UE than SE. Finally, an interaction was found between the encoding performance and the subjective pleasantness judgment for the bilateral PCU, bilateral PCC and left IFG (see Table 2, Figure 4). First of all, the parameter estimates showed that for the PCU, the difference between pleasant and unpleasant judgments was significantly greater for the UE than SE condition. During UE, the pleasant items induced greater BOLD activity in the PCU than the unpleasant items. Contrarily, the parameter estimates showed that for the PCC, the difference between pleasant and unpleasant judgments was greater for the SE than UE condition; during SE, pleasant items induced a greater percentage of MR activity than unpleasant items. Similarly, the parameter estimates revealed that for the IFG, the difference between pleasant and unpleasant judgments was greater for the SE than UE condition; indeed, during SE the unpleasant items induced a greater percentage of signal change than pleasant items.

**Table 1:** **Summary of the regions significantly activated based on the random-effect group analysis for the main effect of encoding performance and pleasantness judgment**.

					MNI coordinates		
Cluster lobe	Brain region	H	BA	*k*	*x*	*y*	*z*	*T*
SE vs. UE
Frontal	Inferior frontal gyrus (Triangularis)	L	45	241	-48	27	14	6.23
	Inferior frontal gyrus (Orbitalis)	L	47		−42	35	−4	4.41
	Precentral gyrus	L	9		−51	9	35	4.04
Temporal	Inferior temporal gyrus	L		83	−48	−63	−7	5.04
	Middle temporal gyrus	L			−51	69	7	4.15
UE vs. SE
Parietal	Precuneus	R	7	146	3	−87	46	5.45
		L	7		−12	−72	49	4.60
	Posterior cingulate cortex	L	23	88	−3	−36	28	4.22
		L	23		−3	−30	28	4.21
Unpleasant vs. Pleasant
Temporal	Inferior temporal gyrus	R	37	86	48	−69	−4	4.50
	Middle temporal gyrus	R	39		48	−89	3.98	3.98

*The main effect of encoding performance includes both contrasts of interest: Successful vs. Unsuccessful and Unsuccessful vs. Successful encoding. The main effect of pleasantness judgment includes one contrast of interest: Unpleasant vs. Pleasant judgments. Statistical threshold for individual voxels was set at p < 0.001 uncorrected with a voxel cluster extent estimated for each of contrast with a Monte Carlo simulation (see “Materials and Methods” Section). The following information is listed for each cluster: cerebral lobe, hemisphere (left, L; right, R), brain region, corresponding Brodmann’s area (BA), MNI coordinates (x, y, z) for the peak activation, number (k) of voxels in each cluster, and statistical value (T scores). Abbreviations: SE, Successful Encoding; UE, Unsuccessful Encoding*.

**Table 2:** **Summary of the regions significantly activated based on the random-effect group analysis for the interaction effect between encoding performance and pleasantness judgment**.

					MNI coordinates		
Cluster lobe	Brain region	H	BA	*k*	*x*	*y*	*z*	*F*
Frontal	Inferior frontal gyrus	L	44/45	69	−48	27	11	14.01
Parietal	Precuneus	R		84	3	−87	42	11.96
	Middle cingulate	R	31	64	6	−27	42	9.47

*Statistical threshold for individual voxels was set at p < 0.001 uncorrected with a voxel cluster extent of 55. The following information is listed for each cluster: cerebral lobe, hemisphere (left, L; right, R), brain region, corresponding Brodmann’s area (BA), MNI coordinates (x, y, z) for the peak activation, number (k) of voxels in each cluster, and statistical value (F scores)*.

## Discussion

The main objective of this fMRI study was to evaluate cerebral activity related to a self-related process, (i.e., the pleasantness judgment) during an incidental memory encoding task, and to evaluate the relationship between the amount of brain activity and the level of task performance (i.e., SE vs. UE). Specifically, we used the pleasantness judgment to predict whether DMN activity would relate to successful or UE given the particular orientation in the pleasantness judgment task. Indeed, the majority of studies posit that the DMN is generally deactivated (i.e., the activation level decreases) while a subject successfully performs an externally-driven task, typically a memory task (e.g., Shrager et al., 2008; Kim, 2011). However, a few studies have indeed reported increased DMN activity related with successful task performance (e.g., Kelley et al., 2002; Leshikar and Duarte, 2012; Maillet and Rajah, 2014). The pleasantness judgment, as used in our study, necessitates increased internal allocation of attention as a self-related subjective decision is required to perform the task. Moreover, given that previous studies revealed that stimuli judged as positive tend to benefit from internally-oriented attention and stimuli judged as negative from the externally-oriented attention and low-level sensory processes (e.g., Mickley and Kensinger, 2008; Ritchey et al., 2011), we expected to show that increased DMN activity would lead to SE for the pleasantly-judged items and to UE for the unpleasantly-judged items. In agreement with these predictions, behavioral performance indicated that participants were significantly more accurate and faster to respond to items rated as pleasant than to items rated as unpleasant. This could suggest that the internally-oriented attention during pleasant items were related with task success.

The ROI analysis based on Fox et al. (2005) showed a significant effect of the Pleasantness judgment on two DMN regions, but no effect on the encoding performance. Specifically, we showed greater involvement of the bilateral mPFC and right LPC during pleasant compared to unpleasant judgments. This is in line with our hypothesis and with behavioral results, as we predicted that pleasant judgments would induce a greater involvement of DMN regions as they depend on internally-oriented, self-relevant thoughts. Considering now the results from the whole brain analysis, only the unpleasant judgment induced significant change in brain activity within the LTC (inferior and middle temporal gyri). These regions, as well as the IFG, were associated with SE. UE recruited the bilateral PCU and the PCC, midlines cortices belonging to the DMN. Asides for the temporal gyri, activity in all of these regions differed according to the type of judgment (pleasant, unpleasant) and the encoding performance (successful, unsuccessful). Thus, the whole brain results suggest posterior DMN modulation by the pleasantness judgment and the encoding performance.

Although different DMN regions were revealed by the ROI and by the whole brain analysis, these results are not contradictory. In the whole brain analysis, an interaction between encoding performance and pleasantness was found for the PCC and the PCU, whereas in the ROI analysis no effects were observed for these regions. The lack of findings for the PCC and PCU in the ROI analysis may have resulted for two reasons. First, as reported by Fox et al. (2005), the PCC and PCU were not dissociated in our analysis, but considered together, as one region in the ROI analysis. Due to the apparent anatomical and functional proximity (i.e., strong covariation in resting state fMRI data) of both structures, most fMRI studies consider the two regions together as a posterior element of the DMN. Nevertheless, a growing number of studies have demonstrated a potential differentiation between PCU- and PCC-based networks. For instance, Whitfield-Gabrieli et al. (2011) have recently shown a dissociation between PCC and PCU activity, their results suggesting that the PCC is more greatly related to self-referential processing whereas the PCU is more greatly related to episodic retrieval. In addition, the localization of these structures based on the MNI coordinates used in the whole brain analysis and those reported by Fox et al. (2005) for the ROI analysis differ greatly and may have contributed to the differing pattern of results observed in this study. In the following sections, we discuss the implications of the results from both types of analyses in terms of the main effects of pleasantness and encoding performance, as well as their interaction.

### Main Effect of Pleasantness Judgment

As illustrated by the ROI analysis, we obtained a significant effect of Pleasantness on the DMN activity, specifically on the mPFC. Although past research has consistently demonstrated the involvement of the mPFC in the emotional evaluation of stimuli during introspective tasks requiring internally-oriented attention (e.g., Gusnard et al., 2001; Phan et al., 2002; Northoff and Bermpohl, 2004; Pallesen et al., 2009; Qin and Northoff, 2011; Maillet and Rajah, 2014), its differential involvement in positive and negative (pleasant or unpleasant) judgments has only scarcely been investigated.

Several studies suggested that the mPFC is involved in emotion processing, regardless of valence (Lane et al., 1997; Phan et al., 2002; LaBar and Cabeza, 2006), suggesting a non-specific involvement of the mPFC in processing emotional information. In the present study, the greater involvement of the mPFC in Pleasant vs. Unpleasant judgments could result from an enhancement of self-referenced introspective processing when a stimulus is “felt” as pleasant. This may include self-projection and planning, as well as an attentional focus on personal semantics and autobiographical representations. Indeed, the mPFC is frequently activated in neuroimaging studies of autobiographical memory (Svoboda et al., 2006; Cabeza and St Jacques, 2007; Bado et al., 2014) and its activation is consistently reported as being related to personal and subjective features of experiencing internal states. Furthermore, the mPFC activity is higher during introspective activities and lower during attention-demanding tasks (Gusnard and Raichle, 2001; Gusnard et al., 2001). Furthermore, the mPFC and its interaction with the medial temporal cortex have been proposed to be involved in memory encoding and retrieval (for a review, see Euston et al., 2012).

Based on the whole brain analysis, we showed that Pleasantness modulates cerebral activity within lateral temporal cortices (inferior and middle gyri), with greater involvement in Unpleasant than in Pleasant judgments. Although temporal cortices are included in DMN, their activity is more weakly correlated with the other DMN regions (Buckner et al., 2008). Activity in the inferior and middle temporal gyri may have been more strongly linked to unpleasant than pleasant judgments because these regions are also related to the dorsal attentional network that is involved in externally-guided cognition (Corbetta and Shulman, 2002), which would have preferentially benefited negative stimuli. As such, the increased activity in the LTC may reflect a higher-level of visual analysis guided toward the perceptive characteristics of the external stimuli (e.g., Smallwood et al., 2012) rather than internally-oriented thought processing.

### Main Effect of Encoding Performance

The whole brain analysis revealed that encoding performance modulated the activity of several regions. Indeed, SE induced fronto-temporal activation, including the inferior and middle temporal gyri and the IFG (pars triangularis and orbitalis), whereas UE induced increased activity in the PCU and PCC (for similar results, see the review by Kim, 2011). The IFG may have been preferentially involved in SE because of its crucial role in the selection, maintenance, organization, and control of incoming information (Ranganath and Knight, 2003; Badre et al., 2005; Badre and Wagner, 2007; Blumenfeld and Ranganath, 2007). The co-activation of the IFG and the inferior/middle temporal gyri may reflect “top-down” attentional processes going from the IFG to the temporal regions in order to maintain and organize the incoming visual information (e.g., Yvert et al., 2012; Perrone-Bertolotti et al., 2014b). This is in line with the findings from several studies that suggest that externally-oriented attention involves a dorsal attentional network comprising of frontal and temporal regions (but also parietal regions not observed here, e.g., Fox et al., 2005). In addition, previous episodic memory studies have revealed a similar frontal and temporal network relation with memory performance (e.g., Schacter and Wagner, 1999; Simons and Spiers, 2003; Kim, 2011). An alternative explanation for the involvement of IFG during SE may be the use of sub-vocalizations (i.e., deliberate inner speech generation) that have been shown to improve cognitive task performance by increasing the focus on the task instruction (for a review, see Perrone-Bertolotti et al., 2014a; Hurlburt et al., 2016).

On the other hand, the relationship between UE and the two DMN regions, PCU and PCC, is in line with past studies demonstrating the role of DMN activation on UE (e.g., Kim, 2011). Both the PCU and PCC are consistently associated with encoding failure (Otten and Rugg, 2001; Wagner and Davachi, 2001; Daselaar et al., 2004, 2009; Kim, 2011; Maillet and Rajah, 2014), despite the type of encoding task employed (Kim, 2011), even if it involves internally-oriented thoughts and introspective processes (e.g., Shrager et al., 2008). Some authors have proposed that the PCU and PCC play a central role in the disengagement of attention from external stimuli to internal thought processes that are often irrelevant to the task at hand (Kircher et al., 2000, 2002; Wagner et al., 2005; Cavanna and Trimble, 2006; Hassabis and Maguire, 2007, 2009; Mason et al., 2007). The encoding failure observed in relation to PCC and PCU activity may therefore result from a disengagement of one’s externally-oriented attention to an internal allocation of attention toward spontaneous thoughts, leading to an insufficient processing of external stimuli (Vannini et al., 2011). This would suggest that the processing of spontaneous internal thoughts and the processing of external input induces competition for attentional resources (Dehaene and Changeux, 2005).

### Interaction Between Pleasantness Judgment and Encoding Performance

Once again, the whole brain analyses revealed that several of the regions involved in the main effect of encoding performance (PCU, PCC, IFG) displayed a different pattern of activation depending on the valence of the pleasantness judgment. Despite the two DMN regions being more greatly involved in UE than SE, they both showed a different pattern of response when considering the pleasantness of the judgments. The PCC was found to be more greatly influenced by the pleasantness judgment for SE than for UE. More specifically, the PCC was more greatly deactivated during SE unpleasant judgments than during SE pleasant judgments. This is coherent with the view that the DMN is deactivated because a decrease in PCC activity was related to an increase in memory performance for unpleasant stimuli only. Although we cannot conclude based on these findings that PCC activity led to increased task performance for pleasant judgments (as an increased activation for SE pleasant judgments was not found), these results suggest that the SE of pleasant and unpleasant stimuli differentially recruit this posterior DMN region. Contrary to the PCC, the PCU was in fact more involved during UE pleasant than during UE unpleasant judgments, suggesting that increased PCU activity during encoding was related to decreased memory performance for pleasant judgments. This might suggest that contrary to our hypothesis, the PCU region of the DMN was task negative for pleasant stimuli, perhaps reflecting increased task-irrelevant thoughts for pleasant compared to unpleasant stimuli. However, no difference in pleasantness judgments was observed for SE, therefore we cannot conclude on whether an increase or decrease in PCU activity is required for a paralleled increase or decrease in successful memory performance for pleasant stimuli. In this study we chose to use the pleasantness judgment as it is a commonly-used incidental encoding task and it encourages the orientation of attention to internal thoughts. However, future studies are needed to fully dissociate relevant from irrelevant thoughts in such tasks.

Finally, the one brain region not associated with the DMN that was found to be more involved in SE than in UE, was the IFG. This region showed greater activity in SE unpleasant than in SE pleasant judgments, although no such difference was observed for the UE conditions. In line with our previous interpretation, the increased involvement of IFG during SE may result from increased sub-vocalization during encoding, that would lead to a greater number of memory traces available upon retrieval. In support of this interpretation, Glotzbach et al. (2011) also found that participants had greater IFG activity when they rated stimuli as negative, and explained these results also in terms of sub-vocalization. It is possible that sub-vocalization is more frequent for unpleasant than for pleasant judgments as they may serve to regulate the negative affect felt in response to the stimulus.

Although our results are generally consistent with our hypotheses, a few limitations should be mentioned. First of all, the conclusions drawn from our whole brain fMRI analyses (threshold dependent) should be interpreted with caution given that the results were obtained with an uncorrected threshold. To minimize potential interpretation errors, we used a voxel cluster extent estimation with Monte Carlo simulation throughout our analyses. Secondly, the PCU region analyzed in our study is more posterior than that observed by other authors such as Fox et al. (2005). According to the dual-attention perspective (Behrmann et al., 2004), the dorsal and verbal parts of the PCU underlie different functions during encoding. The dorsal part of the PCU supports the goal-directed allocation of attention whereas its ventral part reflects self-oriented reflexive attention. Consequently, SE should rely on the dorsal part of the PCU whereas UE should rely on its ventral part. Nevertheless, our results only showed an involvement of the more dorsal part of the PCU during UE, which may be explained by introspective and self-related aspects of the encoding task demands (in relation with pleasantness). Finally, it is also important to highlight that in the present study several types of material (verbal and nonverbal) were used in the incidental memory task. Studies exploring the effect of the cerebral correlates of encoding performances showed different laterality effect on several brain regions according to material (for instance, see Kim, 2011).

## Conclusion

The main objective of this fMRI study was to investigate how the self-related pleasantness judgment influences encoding performance and the DMN activity. Our main results suggest an interaction between the pleasantness judgment and encoding performance on the posterior regions of the DMN, including the PCU and the PCC, and also at the level of the IFG. Furthermore, the mPFC was more involved in Pleasant judgments suggesting that this region is related to self-referential processing. Our results indicate a possible relation between the internal thoughts induced by the pleasantness judgment and the encoding performance, suggesting complex cooperation between DMN and task-successful regions. Results were interpreted in terms of information processing based on an introspective referential and in terms of internally- or external-oriented attention, for pleasant and unpleasant encoded items.

## Author Contributions

MP-B and MB desing research; MP-B, CP and MB performed research; MP-B, MB and CP analyzed data; MP-B, MC, KTR, PH and MB wrote the article. All authors listed, have made substantial, direct and intellectual contribution to the work, and approved it for publication.

## Conflict of Interest Statement

The authors declare that the research was conducted in the absence of any commercial or financial relationships that could be construed as a potential conflict of interest.

## Footnotes

^1^http://marsbar.sourceforge.net/
